# Effectiveness of Radial Extracorporeal Shockwave Therapy in Patients with Acute Low Back Pain—Randomized Controlled Trial

**DOI:** 10.3390/jcm10235569

**Published:** 2021-11-26

**Authors:** Tobias Lange, Niklas Deventer, Georg Gosheger, Lukas P Lampe, Sebastian Bockholt, Albert Schulze Boevingloh, Tobias L Schulte

**Affiliations:** 1 Department of Orthopaedics and Trauma Surgery, St. Josef-Hospital, Ruhr-University Bochum, 44791 Bochum, Germany; tobias.schulte@klinikum-bochum.de; 2 Department of Orthopaedics and Tumour Orthopaedics, Muenster University Hospital, 48149 Muenster, Germany; Niklas.Deventer@ukmuenster.de (N.D.); georg.gosheger@ukmuenster.de (G.G.); lukas.lampe@ukmuenster.de (L.P.L.); sebastian.bockholt@ukmuenster.de (S.B.); albert.schulzeboevingloh@ukmuenster.de (A.S.B.)

**Keywords:** low back pain, acute, radial shockwave therapy, randomized controlled trial (RCT), clinical outcome, spine

## Abstract

The aim of this study was to investigate the effect of radial extracorporeal shockwave therapy (rESWT) primarily on acute lumbar back pain (aLBP), and secondarily on physical function and quality of life. This randomized, placebo-controlled, single-blinded trial with 12-week follow-up (FU) randomized 63 patients with aLBP 1:1 into two groups receiving either rESWT (intervention) or sham rESWT (placebo) with a manipulated shockwave head not delivering any shockwaves. Both, rESWT and sham procedure were carried out eight times for four weeks. Both groups received additional analgesics and physiotherapy twice a week. Primary patient-reported outcome measure (PROM) was the visual analogue scale for aLBP (VAS-LBP). Secondary PROMs included the Oswestry disability index (ODI), Roland and Morris Disability Questionnaire (RDQ), EuroQol EQ-5D-3L, and the Beck Depression Index (BDI-II). Primary endpoint was a between-arm comparison of mean changes in VAS-LBP from baseline to final FU. At randomization, there were no differences between the two groups in relation to age and PROMs. Both groups showed significant improvement in all PROMs at final FU. VAS-LBP declined by 60.7% (*p* < 0.001) in the intervention and by 86.4% (*p* < 0.001) in the sham group. The intervention group showed significantly less pain relief after 4 and 12 weeks. The EQ-5D submodality pain showed significantly inferior results for the intervention (1.5 (0.58)) compared to the sham group (1.1 (0.33)) (*p* < 0.014) after eight weeks. No significant intergroup differences were observed for RDQ, ODI or BDI-II. Additional rESWT alongside conventional guideline therapy in aLBP does not have any significant effects on pain intensity, physical function, or quality of life. To the best of our knowledge, this is the first study with a high level of evidence reporting the efficacy of rESWT in aLBP treatment and will be a future basis for decision-making.

## 1. Introduction

Acute low back pain (aLBP), with a lifetime prevalence of up to 85% among 18–74-year-olds, is one of the most common types of pain associated with the musculoskeletal system [1,2,3]. The costs of treatment for musculoskeletal disease have been increasing dramatically in Western countries in recent years. In 2008, they accounted for 11.2% of total health-care costs in Germany [4]. The German National Care Guidelines on “nonspecific low back pain” list nondrug therapies and assesses the evidence of their efficacy in the treatment of acute nonspecific low back pain [5]. Radial extracorporeal shockwave therapy (rESWT) is not mentioned in the guidelines, due to the lack of scientific data for it. However, it is often used as a treatment option in private practice and has been reported to have positive treatment results in relation to chronic pain [6,7,8,9,10].

Initially, ESWT found its way into medicine as a treatment option for urolithiasis (focused ESWT) [11]. Extracorporeal shock waves are also used as a treatment for musculoskeletal painful diseases such as bony pseudarthrosis [12], achilles tendinopathy, or plantar fasciitis [13]. Three different mechanisms of ESWT are existent: focused, defocused, and radial [14].

It has not yet been investigated whether rESWT is also effective in the treatment of nonspecific aLBP. The aim of this study was to investigate the effects of rESWT on nonspecific aLBP, physical function, and quality of life.

It was hypothesized that rESWT is more effective in improving acute low back pain and physical function than sham rESWT, each combined with a standardized analgesic and physiotherapeutic regimen.

## 2. Materials and Methods

**Figure 1:**
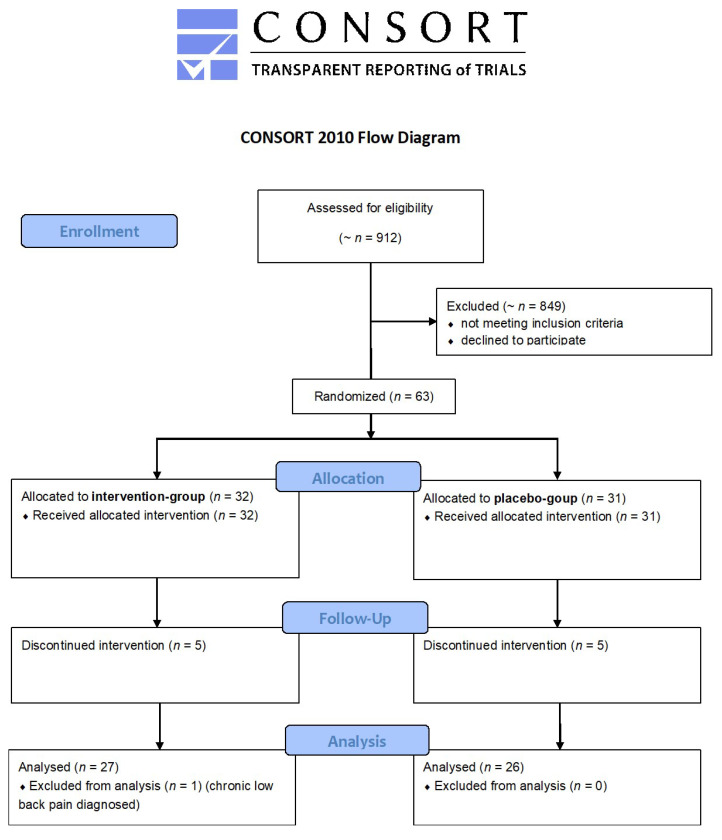
Consolidated standards of reporting trials (CONSORT) 2010 flow diagram for patient selection.

In this randomized controlled trial (RCT), all patients with aLBP who visited the outpatient department in our hospital over the course of one year (*n* = 912) were assessed for eligibility. Those who did not meet the inclusion criteria (age 18–65 years, onset of aLBP < 3 months previously) or had at least one exclusion criterion (relevant leg pain, sensorimotor deficiency, previous spinal surgery, scoliosis > 10°, or traumatic, cancerous, infectious, or psychiatric diseases, known osteoporosis, previous rESWT, oral anticoagulant therapy) and those who declined to participate in the trial were excluded (Figure 1). A total of 63 patients with aLBP were finally enrolled after providing informed consent.

All the patients were blinded, whereas the principal investigator was not. The blinded patients were then randomly assigned 1:1 into two groups by the principal investigator, using the sealed opaque envelope technique [15]. Once a patient has consented to enter the trial, one of the sequentially numbered envelopes/containers was opened and the patient was then offered the allocated treatment regimen. Group 1 (intervention, *n* = 27) received shockwave therapy (rESWT). The EMS Swiss DolorClast^®^ Classic device with Evo Blue handpiece (EMS Electro Medical Systems S.A., Nyon, Switzerland) was used to administer rESWT, with the patients in the prone position. Low-energy impulses were applied to determine points of maximum tenderness (PMTs). Following this, 400 shockwave impulses (8 Hz) at an energy flux density of up to 0.28 mJ/mm^2^ were delivered to all PMTs using a 36-mm applicator head. Finally, all paravertebral lumbar muscles were again treated with 500 low-energy impulses (10 Hz) of up to 0.18 mJ/mm^2^.

In the sham group (placebo, *n*=26), a second shockwave head was manipulated by EMS Electro Medical Systems that did not deliver any shockwaves. The “treatment” procedure in the sham group remained the same as in the intervention group (rESWT). The treatment procedure lasted for 15 min. Both groups received treatment twice a week over a period of four weeks (a total of eight rESWT or sham rESWT sessions). Additionally, all patients received oral analgesics (ibuprofen 600 mg 3 q/day). Three patients (two in the control and one in the intervention group) with individual contraindications for ibuprofen did receive different analgesics. Furthermore, physiotherapy was prescribed for all patients twice a week, and they were followed up for 12 weeks.

The patients randomly came to the clinic when their acute low back pain first appeared. After enrollment in the study, all participants were treated and interviewed at individual times. Intercommunication between the intervention and sham group was therefore fundamentally ruled out.

A preliminary test was added after the start of the trial, as it was still uncertain whether rESWT might be a painful therapeutic option in aLBP. In the preliminary test, five patients were not randomized but were allocated to the intervention group and received rESWT. These five patients were obviously still blinded. It became apparent that administering rESWT with the technical parameters selected is a totally painless procedure.

An a priori power analysis was performed, based on comparable studies on low back pain, and showed a minimal clinically important difference (MCID) of 14.6–20.0 mm on the visual analogue scale for low back pain (VAS-LBP, range: 0–100 mm) [7,16,17,18]. An MCID of 20 mm was defined on the VAS-LBP to make the planned study as statistically strong as possible. Using a power of 90% and an alpha level of 0.025, it was determined that 56 patients in total, 28 patients in each group, were needed to detect a difference of 20 mm on the average VAS-LBP between the intervention group and sham group (software: G*power, version 3.1.9.3, Duesseldorf, Germany). However, expecting a dropout of approximately 5 patients, we ultimately included 63 patients in this study.

The primary patient-reported outcome parameter was the pain intensity of aLBP, measured using the VAS-LBP [19]. Additionally, secondary physical function outcome parameters were reported by measuring the Oswestry disability index (ODI), the Roland and Morris Disability Questionnaire (RDQ), and EQ-5D-3L. The possibility of bias due to depressive disorders was addressed using the Beck Depression Index (BDI-II). Depressive disorders or symptoms are well known to be an important factor modulating the perception of pain [20]. All data were obtained initially prior to treatment (t_0_) and after 1, 2, 3, 4, 6, 8, and 12 weeks.

Statistical analysis was performed using IBM SPSS Statistics, version 26.0 (IBM Corporation, Armonk, NY, USA). All outcome measures were analyzed in accordance with the manual for the respective questionnaire. For reliability analysis, Cronbach’s alpha was calculated to assess the internal consistency of the questionnaires’ subscales.

Data were then pooled by group to calculate the mean (standard deviation (SD)) for each outcome parameter. After testing for gaussian distribution using the Kolmogorov–Smirnov test, analysis of repeated measures with post hoc analysis according to Bonferroni was used for data comparison. The significance level was set at *p* < 0.05.

## 3. Results

A total of 53 of the 63 enrolled patients were eligible for statistical analysis. Ten patients were lost to follow-up and did not complete all of the surveys, due to a loss of interest in the study after pain recovery. Since some of the patients missed appointments, the 27 patients in the intervention group (mean age 38.7 (12.91) years; 11 men, 16 women) received a mean of 7.4 rESWT treatments, and the 26 patients in the sham group (mean age 40.4 (11.61) years; nine men, 17 women) received a mean of 7.0 sham rESWT treatments.

Before therapy, there were no significant differences between the two groups in the VAS-LBP, RDQ, ODI, EQ-5D-3L, or BDI-II scores (Table 1).

**Figure 2:**
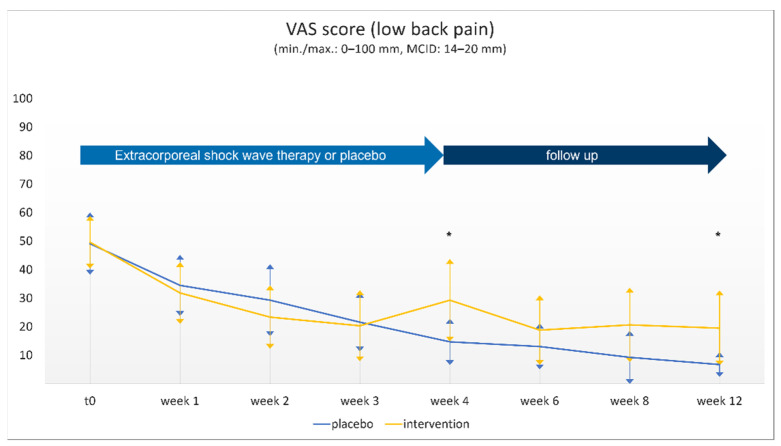
The pain level according to the visual analogue scale for low back pain (VAS-LBP; mean (SD)) for the two groups (intervention vs. placebo) at the eight different time points. Asterisks (*) indicate a significant difference (*p* < 0.05).

Intragroup comparisons showed significant improvement in all parameters recorded over the 12-week period (Table 1). Pain intensity (VAS-LBP) declined from 49.6 (18.47) to 19.5 (26.36) (*p* < 0.001) in the intervention group and from 49.1 (21.81) to 6.7 (8.74) (*p* < 0.001) in the sham group. The intervention group showed significantly less pain relief after 4 and 12 weeks (Figure 2).

**Figure 3:**
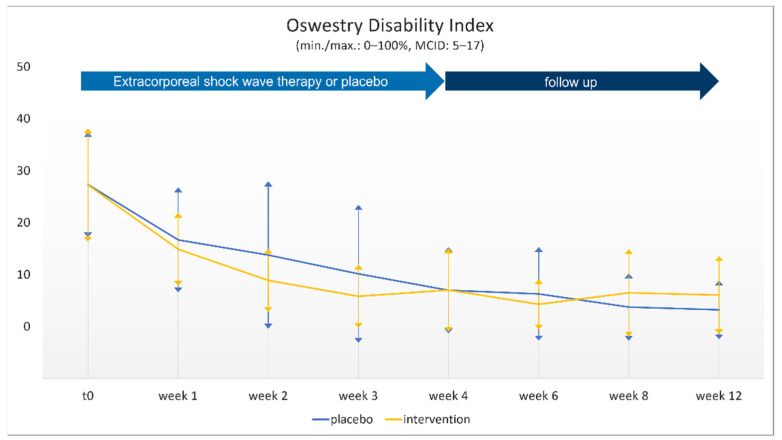
Physical function according to the Oswestry disability index (ODI; mean (SD)) for the two groups (intervention vs. placebo) at the eight different time points.

The EQ-5D-3L submodality “pain” showed significantly inferior results for the intervention group (1.5 (0.58)) in comparison with the sham group (1.1 (0.33); *p* < 0.014) after eight weeks (Table 1). Although the RDQ showed superiority for rESWT in comparison with the sham group after two and three weeks, there was a trend at the final follow-up towards inferiority of rESWT in the intervention group (intervention 2.0 (0.33) vs. sham 0.8 (0.80)). No differences were observed for ODI (Figure 3) or BDI-II.

For reliability analysis, Cronbach’s alpha was calculated to assess the internal consistency of the questionnaires’ subscales. The internal consistency of EQ-5D-3L, ODI, RDQ and BDI-II is overall satisfying, with Cronbach’s alpha varying between 0.736 and 0.891.

## 4. Discussion

Acute low back pain, which is mostly managed in primary care, is a common cause of disability all over the world [2]. To our knowledge, this is the first study analyzing the effectiveness of acute low back pain treatment using rESWT in comparison with a sham treatment. The results suggest that rESWT combined with physiotherapy and analgesia was not superior to analgesia and physiotherapy alone, in relation to pain intensity and physical function.

In general, this result is contrary to previous studies that had investigated the effectiveness of shock wave therapy for chronic lumbar back pain. Han et al. found that pain intensity decreased significantly when ESWT was administered to patients with chronic low back pain. Lee et al. also reported that a combination of ESWT and physiotherapy was more effective in relation to VAS scores and dynamic balance activity in comparison with an exercise program and physical therapy [21,22]. Notarnicola et al. conducted a clinical trial including 30 patients suffering from chronic low back pain, who were randomly assigned to a shockwave therapy group or a rehabilitation exercise group [10]. The authors reported a significant improvement in pain intensity and physical function after one and three months in the shockwave therapy group in comparison with the control group. However, by choosing a control group who received only standard rehabilitation exercises, they did not control for a possible placebo effect in the same way as the present study did. The results of a review by Seco et al. did not support the use of shockwave therapy in patients with chronic low back pain and leg pain. They conclude that the available evidence does not support the effectiveness of shock wave therapy in low back pain and that high-quality RCTs are needed to assess their efficacy vs. appropriate sham procedures [8].

In the present study, intragroup comparisons showed significant decreases in the VAS level, ODI, RDQ, and EQ-5D-3L in both the intervention group and the sham group at the end of the 12-week follow-up period. However, intergroup comparison revealed that these decreases were larger in the sham group than in the rESWT group. Wang reported that extracorporeal shock waves stimulate the expression of angiogenesis-related growth factors, including endothelial nitric oxide synthase (eNOS), vascular endothelial growth factor (VEGF), and proliferating cell nuclear antigen (PCNA), which induce neovascularization and improve blood flow in tissues, stimulating healing processes in inflammatory conditions in tendons, bones, and surrounding tissues, and resulting in pain relief [23]. These growth factors increased as early as one week after the start of ESWT and lasted for about eight weeks, whereas neovascularization took place between four and twelve weeks after the start of ESWT [24]. The effects involved were mostly medium-term and long-term and may be of value in the treatment of chronic low back pain, as Lee et al. reported. However, they do not appear to play a major role in acute low back pain, as the present study did not observe any benefit of rESWT in relation to VAS level or physical function parameters. Therefore, a too short follow-up period could have been decisive and is therefore one of the limitations of this study.

Although there is some evidence suggesting that the measurement properties of visual analogue scales are superior to those of other patient-reported outcome measures such as the numeric rating scale or brief pain inventory, the VAS is widely used in studies of low back pain. The MCID used in VAS surveys (0–100 mm) varies between 14 and 20 mm, depending on different studies [7,16,18]. In the present study, intragroup changes in the VAS over time showed values of about 30–40 mm in both groups, and they must therefore be considered clinically relevant. Consequently, the therapy must be rated as effective in terms of reducing low back pain. By contrast, the intergroup analysis showed a difference of 12.8 mm at the final follow-up, even to the disadvantage of the intervention group, and missed the MCID limit of about 20 mm. This leads to the conclusion that the study did not demonstrate any superiority of rESWT over the conventional therapy.

No complications were observed in the patients throughout the study, a finding that is in line with literature reports [25].

One limitation of the present study was that, although the aim was to enroll 56 patients and the enrollment target was met (*n* = 63), 10 patients dropped out during the ongoing study. The statistical analysis is therefore only based on 53 patients. However, it is unlikely that this small deviation could have led to an inconsistent result. Nevertheless, further research arising from the single-center data presented here is therefore needed in order to verify the findings in larger cohort studies based on a multicenter RCT.

A further limitation could be seen in the used shockwave technique. The use of radial ESWT instead of focused or defocused ESWT should be considered when assessing the effectiveness of ESWT as a treatment technique [14]. The present study of course only shows that radial ESWT does not have any additional positive effects in the treatment of acute low back pain in comparison with standard treatment. These findings are not directly transferable to focused ESWT and need to be evaluated in further research. However, Schmitz et al. have already reported that there is no scientific evidence in favor of either rESWT or focused ESWT [26]. The technical parameters for rESWT selected in the present study, such as the number of shockwave impulses (400–500 at 8–10 Hz), energy flux density (0.18–0.28 mJ/mm^2^), and frequency of rESWT application (twice per week), are further possible variables that could be altered in future studies [27]. In their systematic review, Schmitz et al. concluded that applying insufficient energy may adversely affect the outcome of ESWT in calcifying tendinitis of the shoulder, plantar fasciopathy, and Achilles tendinopathy [26]. Future studies will need to clarify whether this finding also applies to acute lumbar back pain; it might explain the surprising inferiority of rESWT in comparison with sham therapy in the present study. As a consequence, a change in the technical parameters of rESWT could increase the effectiveness of this technique with respect to the therapy of acute low back pain and should be investigated in large RCTs.

## 5. Conclusions

In conclusion, systematic comparative studies on this clinically relevant topic are still very rare. This is the first randomized, placebo-controlled trial to analyze the efficacy of rESWT in the treatment of aLBP. From the clinical point of view, rESWT is already used in practice, but without sufficient scientific data. The present trial was conducted in order to help fill this information gap, focusing on whether rESWT might be an option in the treatment of aLBP. This approach is unique in the literature.

Both study groups showed significant improvements in VAS, EQ-5D-L, ODI and RDQ throughout the therapy. However, additional administration of radial shockwave therapy in patients with acute lumbar back pain did not show any better or earlier reduction in pain intensity or improvement in physical function, in comparison with sham treatment. Larger multicenter studies are desirable to confirm the findings of this randomized controlled trial.

## Figures and Tables

**Table 1:** Results for VAS-LBP, EQ-5D-3L, ODI, RDQ and BDI-II.

Questionnaire	t_0_	Week 1	Week 2	Week 3	Week 4	Week 6	Week 8	Week 12
	mean ± SD							
VAS-LBP								
placebo	49.1 ± 21.81	34.5 ± 21.42	29.3 ± 25.22	21.6 ± 20.62	14.7 ± 16.25	13.1 ± 16.11	9.2 ± 18.66	6.7 ± 8.74
intervention	49.6 ± 18.47	31.8 ± 21.74	23.4 ± 22.26	20.3 ± 24.88	29.3 ± 28.90	18.8 ± 24.38	20.6 ± 26.14	19.5 ± 26.36
*p*	0.933	0.670	0.377	0.848	* **0.029**	0.322	0.079	* 0.026
EQ5D-3L								
mobility								
placebo	1.4 ± 0.51	1.3 ± 0.44	1.2 ± 0.43	1.1 ± 0.33	1.0 ± 0.34	1.1 ± 0.27	1.0 ± 0.00	1.0 ± 0.00
intervention	1.4 ± 0.50	1.1 ± 0.37	1.1 ± 0.36	1.0 ± 0.28	1.2 ± 0.36	1.1 ± 0.30	1.1 ± 0.20	1.1 ± 0.20
*p*	1.000	0.088	0.115	0.348	0.248	0.646	0.163	0.146
self care								
placebo	1.1 ± 0.28	1.0 ± 0.20	1.1 ± 0.27	1.0 ± 0.00	1.0 ± 0.20	1.0 ± 0.00	1.0 ± 0.00	1.0 ± 0.00
intervention	1.1 ± 0.28	1.0 ± 0.19	1.0 ± 0.00	1.0 ± 0.00	1.0 ± 0.00	1.0 ± 0.00	1.0 ± 0.00	1.0 ± 0.00
*p*	1.000	0.934	0.147	-	0.322	-	-	-
activity								
placebo	1.6 ± 0.51	1.3 ± 0.47	1.4 ± 0.57	1.2 ± 0.37	1.0 ± 0.20	1.1 ± 0.33	1.0 ± 0.20	1.0 ± 0.20
intervention	1.5 ± 0.51	1.2 ± 0.40	1.1 ± 0.32	1.1 ± 0.29	1.1 ± 0.27	1.1 ± 0.33	1.2 ± 0.37	1.2 ± 0.41
*p*	0.580	0.335	* 0.035	0.455	0.561	1.000	0.175	0.075
pain								
placebo	2.0 ± 0.35	1.9 ± 0.46	1.7 ± 0.55	1.4 ± 0.51	1.4 ± 0.50	1.3 ± 0.49	1.1 ± 0.33	1.2 ± 0.44
intervention	2.0 ± 0.32	1.8 ± 0.44	1.6 ± 0.53	1.3 ± 0.49	1.5 ± 0.50	1.4 ± 0.49	1.5 ± 0.50	1.5 ± 0.48
*p*	0.662	0.465	0.350	0.454	0.412	0.779	* 0.014	0.113
anxiety								
placebo	1.0 ± 0.20	1.0 ± 0.00	1.0 ± 0.00	1.0 ± 0.00	1.0 ± 0.20	1.0 ± 0.00	1.0 ± 0.00	1.0 ± 0.00
intervention	1.1 ± 0.33	1.0 ± 0.19	1.0 ± 0.19	1.0 ± 0.00	1.0 ± 0.20	1.0 ± 0.00	1.0 ± 0.20	1.0 ± 0.20
*p*	0.307	0.361	0.331	-	0.163	-	0.332	0.312
EQ-VAS								
placebo	67.4 ± 22.45	71.6 ± 20.99	75.3 ± 22.21	79.5 ± 21.61	78.8 ± 21.37	87.2 ± 13.03	91.8 ± 8.32	90.2 ± 14.25
intervention	69.8 ± 15.72	79.1 ± 12.18	83.3 ± 13.78	83.9 ± 20.30	83.7 ± 16.51	86.5 ± 16.50	84.8 ± 17.87	85.4 ± 18.96
*p*	0.669	0.122	0.120	0.471	0.368	0.867	0.081	0.318
ODI								
placebo	27.3 ± 10.13	16.7 ± 10.12	13.8 ± 14.18	10.2 ± 13.28	7.0 ± 8.32	6.3 ± 9.01	3.8 ± 6.51	3.2 ± 5.66
intervention	27.3 ± 10.91	14.9 ± 7.05	8.9 ± 6.11	5.9 ± 6.09	7.0 ± 8.07	4.3 ± 4.89	6.5 ± 8.42	6.1 ± 7.51
*p*	0.987	0.453	0.108	0.139	0.987	0.315	0.191	0.127
RDQ								
placebo	7.9 ± 4.14	6.0 ± 4.67	5.1 ± 4.92	3.2 ± 3.78	2.0 ± 2.49	1.7 ± 2.28	1.0 ± 1.27	0.8 ± 0.80
intervention	7.0 ± 4.17	3.6 ± 2.33	2.9 ± 2.41	1.9 ± 2.35	2.0 ± 2.29	1.7 ± 1.69	2.1 ± 2.79	2.0 ± 3.31
*p*	0.482	* 0.019	* 0.041	0.138	1	0.945	0.065	0.107
BDI-II								
placebo	5.4 ± 5.70	3.9 ± 4.59	4.0 ± 5.24	2.4 ± 4.36	2.0 ± 3.48	1.7 ± 3.19	1.2 ± 2.87	1.4 ± 3.01
intervention	5.2 ± 4.72	3.7 ± 4.11	2.3 ± 3.46	1.4 ± 3.45	1.8 ± 3.63	0.9 ± 2.14	1.4 ± 3.17	2.0 ± 5.16
*p*	0.874	0.856	0.177	0.345	0.85	0.263	0.797	0.615

Presented as mean ± SD (standard deviation) and *p* values, each for eight different time points (t0 and weeks 1–12); Bold, “*” indicate a significant difference (*p* < 0.05).
